# Understanding anti‐TNF treatment failure: does serum triiodothyronine‐to‐thyroxine (T3/T4) ratio predict therapeutic outcome to anti‐TNF therapies in biologic‐naïve patients with active luminal Crohn's disease?

**DOI:** 10.1111/apt.17089

**Published:** 2022-06-29

**Authors:** Simeng Lin, Neil Chanchlani, Isabel Carbery, Malik Janjua, Rachel Nice, Timothy J. McDonald, Claire Bewshea, Nicholas A. Kennedy, Tariq Ahmad, Christian P. Selinger, James R. Goodhand, PANTS consortium

**Affiliations:** ^1^ Gastroenterology Royal Devon and Exeter NHS Foundation Trust Exeter UK; ^2^ Exeter Inflammatory Bowel Disease and Pharmacogenetics Research Group University of Exeter Exeter UK; ^3^ Leeds Gastroenterology Institute, Leeds Teaching Hospitals NHS Trust Leeds UK; ^4^ The Leeds Institute of Research at St James' University of Leeds Leeds UK; ^5^ Biochemistry, Exeter Clinical Laboratory International Royal Devon and Exeter NHS Foundation Trust Exeter UK

**Keywords:** Crohn’s disease, IBD, low T3 syndrome, low T3/T4 ratio, non‐thyroidal illness syndrome, PANTS, sick euthyroid syndrome, T3, T4, TSH

## Abstract

**Background:**

During illness, adaptations of the hypothalamic–pituitary‐thyroid axis reduce energy expenditure, protein catabolism and modulate immune responses to promote survival. Lower serum free triiodothyronine‐to‐thyroxine (fT3/fT4) ratio has been linked to non‐response to treatment in a range of diseases, including in biologic‐treated patients with inflammatory bowel disease.

**Aim:**

To assess whether baseline serum fT3/fT4 ratio predicted primary non‐response (PNR) and non‐remission to infliximab and adalimumab in patients with Crohn's disease

**Methods:**

Thyroid function tests were undertaken in stored serum from biologic‐naïve adult patients with active luminal Crohn's disease immediately prior to treatment with infliximab (427 originator; 122 biosimilar) or adalimumab (448) in the Personalised Anti‐TNF Therapy in Crohn's Disease study (PANTS).

**Results:**

Baseline median [IQR] fT3/fT4 ratios were lower in women than men (0.30 [0.27–0.34] vs 0.32 [0.28–0.36], p < 0.001), in patients with more severe inflammatory disease, and in patients receiving corticosteroids (0.28 [0.25–0.33] vs. 0.32 [0.29–0.36], *p* < 0.001). Multivariable logistic regression analysis demonstrated that fT3/fT4 ratio was independently associated with PNR at week 14 (odds ratio [OR] 0.51, 95% confidence interval [CI] 0.31–0.85, *p* = 0.009), but not non‐remission or changes in faecal calprotectin concentrations at week 54. The optimal threshold to determine PNR was 0.31 (area under the curve 0.57 [95% CI 0.54–0.61], sensitivity 0.62 [95% CI 0.41–0.74], and specificity 0.53 [95% CI 0.42–0.73]).

**Conclusions:**

Lower baseline serum fT3/fT4 ratio was associated with female sex, corticosteroid use and disease activity. It predicted PNR to anti‐TNF treatment at week 14, but not non‐remission at week 54.

## Graphical abstract

In 995 patients with Crohn’s disease from the PANTS study, lower baseline serum fT3/fT4 ratio was independently associated with female sex, higher inflammatory burden at baseline, and baseline corticosteroid use, and predicted primary non‐response to anti‐TNF therapy at week 14, but not non‐remission or change in faecal calprotectin concentrations at week 54. Overall, however, the diagnostic accuracy of baseline fT3/fT4 ratio to predict primary nonresponse to anti‐TNF treatment was modest, limiting its clinical utility.

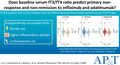

## 1 BACKGROUND

Ulcerative colitis (UC) and Crohn's disease are archetypal relapsing and remitting immune‐mediated inflammatory diseases of the gut that affect about 1% of western populations.^1^, ^2^ Active disease is characterised by gastrointestinal inflammation, malnutrition, reduced quality of life and increased rates of depression.

During acute illnesses, adaptations of the hypothalamic–pituitary‐thyroid axis reduce energy expenditure, protein catabolism and modulate immune processes to promote survival.^3^, ^4^ Most, if not all, critically ill patients have low serum‐free triiodothyronine (fT3) and low‐normal‐free thyroxine (fT4) levels without a compensatory rise in thyroid‐stimulating hormone (TSH).^3^, ^4^ This so‐called non‐thyroidal illness‐, sick euthyroid‐ or low T3‐ syndrome, has been consistently linked to illness severity and outcome, including with COVID‐19.^4^, ^5^ Similar observations have also been made in patients with chronic diseases including heart failure, renal failure, neurological dysfunction and inflammatory bowel disease (IBD).^6^, ^7^, ^8^, ^9^

The anti‐TNF monoclonal antibodies, infliximab and adalimumab, are the most frequently prescribed biologic medications and have transformed the management of IBD. In Crohn's disease, successful treatment leads to mucosal healing, reduced surgeries and improvements in quality of life.^10^, ^11^ Regrettably, however, anti‐TNF treatment failure is common. About one‐quarter of patients experience primary non‐response (PNR) and one‐third of initial responders lose response, such that only one‐third of patients are in remission at the end of a year.^12^

The biology of non‐response is complex, but being able to predict who will fail anti‐TNF therapy could help prompt concomitant immunomodulator use, anti‐TNF dose optimisation and biologic sequencing. Multiple patient, disease, and drug‐related factors have been implicated in anti‐TNF treatment failure, but few studies have been adequately powered to define their relative effects, interactions and impact on drug and anti‐drug antibody levels.^13^ In the PANTS study, we showed that obesity, cigarette smoking, higher baseline markers of disease activity, anti‐TNF monotherapy and the development of antidrug antibodies are associated with low drug levels and anti‐TNF treatment failure.^12^ Carriage of the HLADQA1*05 allele confers a two‐fold risk of developing antibodies to anti‐TNF treatment.^14^

Recent data reported by Bertani et al. showed that low serum triiodothyronine‐to‐thyroxine ratios (fT3/fT4) at initiation of infliximab or vedolizumab therapy predicted poor endoscopic outcomes at 54 weeks in a mixed cohort of patients with UC and Crohn's disease.^15^

We sought to assess whether baseline serum fT3/fT4 ratio predicted PNR and non‐remission to infliximab and adalimumab in patients with Crohn's disease.

## 2 METHODS

### 2.1 Study design

The Personalised Anti‐TNF Therapy in Crohn's Disease study (PANTS) is a UK‐wide, multicentre, prospective observational cohort reporting the treatment failure rates of the anti‐TNF drugs infliximab (originator, Remicade [Merck Sharp & Dohme, UK] and biosimilar, CT‐P13 [Celltrion, South Korea]) and adalimumab (Humira [Abbvie, USA]) in anti‐TNF‐naïve patients with active luminal Crohn's disease.^12^

Patients were recruited at the time of first anti‐TNF exposure between February 2013 and June 2016 and studied for 12 months or until drug withdrawal (Table S1). Eligible patients were aged ≥6 years with objective evidence of active luminal Crohn's disease involving the colon and/or small intestine. Exclusion criteria included prior exposure to, or contraindications for the use of, anti‐TNF therapy.

The choice of anti‐TNF was at the discretion of the treating physician and prescribed according to the licensed dosing schedule. Study visits were scheduled at first dose, post‐induction (week 14), and at weeks 30 and 54. Additional visits were planned for infliximab‐treated patients at each infusion and for both groups at treatment failure or exit.

For this analysis, we included adult patients over the age of 17 years only because of limited or exhausted stored serum in paediatric patients. Patients who were treated with endocrine‐related medications that may have affected the hypothalamic–pituitary‐thyroid axis, including thyroxine, carbimazole, growth hormone and testosterone, or who had evidence of possible primary hypothyroidism or Grave's disease, were excluded.

### 2.2 Outcomes

Treatment failure endpoints were PNR at week 14, non‐remission at week 54 and adverse events leading to drug withdrawal. We used composite endpoints defined using the Harvey Bradshaw Index (HBI),^16^ corticosteroid use and C‐reactive protein (CRP).

*PNR*: exit prior to week 14 for treatment failure (including resectional IBD surgery) or corticosteroid use at week 14 (new prescriptions or failure to taper). Patients who exhibited both a failure of CRP to fall to ≤3 mg/L or by 50% from baseline (week 0) and failure of HBI to fall to ≤4 or by 3 points were also classified as PNR.

*Grey zone (intermediate between PNR and response)*: CRP falls to ≤3 mg/L or by 50% from baseline (Week 0) or HBI falls to ≤4 or by 3 points from baseline (but not both).

*Response:* both CRP falls to ≤3 mg/L or by 50% from baseline (Week 0) and HBI falls to ≤4 or by 3 points from baseline.

*Remission*: CRP of ≤3 mg/L and HBI of ≤4 points, no ongoing corticosteroid therapy and no exit for treatment failure.

*Non‐Remission* was assessed at week 54 and defined as either CRP of >3 mg/L or HBI of >4 points, ongoing corticosteroid therapy or exit for treatment failure.

We defined corticosteroid therapy for the purposes of non‐remission and PNR as any systemic therapy, including prednisolone and budesonide, either oral or intravenous. We included use of corticosteroids for other conditions, but excluded use single pre‐biologic infusion dosing with hydrocortisone.

*Patients excluded from effectiveness analysis:* Three groups of patients were excluded from our effectiveness analyses.^12^ First, patients with stomas, because the HBI is not validated in this patient group; second, patients who were recruited into the study with normal calprotectin and CRP concentrations at pre‐screening and during the first visit; third, patients for whom the only indication for anti‐TNF treatment was perianal disease.

*Exit:* Patients exited the study when they stopped anti‐TNF therapy or had an intestinal resection. Patients who exited the study for treatment failure were deemed to be in non‐remission for subsequent time points. Patients who exited the study for loss to follow‐up, withdrawal of consent, or elective withdrawal of drug, including for pregnancy, were censored at the time of study exit and excluded from the denominator for subsequent analyses.

### 2.3 Clinical and laboratory variables

At baseline, sites recorded demographic data (sex, ethnicity, body mass index [BMI]), smoking status, age at diagnosis, disease duration, Montreal classification,^17^ prior medical and drug history and previous Crohn's disease‐related surgeries. At every visit, disease activity score, weight, current therapy and adverse events were recorded.

Blood and stool samples were processed through the central laboratory at the Royal Devon and Exeter NHS Foundation Trust (https://www.exeterlaboratory.com/) for haemoglobin, white cell count, platelets, serum albumin, CRP, anti‐TNF drug and anti‐drug antibody concentrations and faecal calprotectin, respectively. All analysis were carried out on the Cobas 801 module of the Cobas 8000 automated platform (Roche Diagnostics).

Serum TSH, triiodothyronine and thyroxine levels were measured on stored baseline samples between 30 November 2021 and 7 January 2022. The Roche Elecsys TSH immunoassay is an electrochemiluminescence assay that sandwiches TSH between biotinylated and ruthenium‐complexed TSH‐specific monoclonal antibodies.^18^ The local reference range is 0.27–4.2 μIU/ml. The Roche Elecsys T3 and T4 electrochemiluminescence assays are competitive immunoassays. The reference ranges are 3.1–6.8 pmol/L and 1.2–22.0 pmol/L, respectively.

### 2.4 Study size and statistical methods

The assumptions underlying the PANTS sample size calculation have been reported previously.^12^ Herein, we included all adult patients who had sufficient stored serum at baseline for analysis who had outcome data at week 14.

Statistical analyses were undertaken in R 4.1.2 (R Foundation for Statistical Computing, Vienna, Austria). All tests were two tailed, and *p*‐values <0.05 were considered significant. We included patients with missing clinical variables in analyses for which they had data and have specified the denominator for each variable. Continuous data are reported as median and interquartile range and discrete data as numbers and percentages.

We performed univariable analyses using Fisher's exact and Mann–Whitney U tests to identify differences in baseline characteristics between infliximab‐ and adalimumab‐treated patients, and to determine categorical factors associated with fT3/fT4 ratio and predefined outcomes. Spearman's rank correlation was used to determine continuous factors, including faecal calprotectin, associated with fT3/fT4 ratio. Multivariable logistic regression models were used to identify factors independently associated with PNR at week 14 and remission at week 54. Variables identified in the PANTS study as associated with each outcome were included in the model.^12^ For infliximab treatment, this included older age, smoking, immunomodulator use at baseline and albumin; and for adalimumab‐treatment, older age and BMI.

Youden's formula^19^ was used to determine the optimal fT3/fT4 ratio cut‐off to predict PNR, and receiver operator characteristic curves and area under the curve analyses with bootstrapping were used to estimate the diagnostic accuracy of the model. We performed sensitivity analyses restricting the cohort to patients not treated with corticosteroids at baseline and in line with the inclusion criteria in the study by Bertani et al., to those over 60 years.

### 2.5 Role of the funding source

PANTS is an investigator‐led study funded by the research charities CORE, Crohn's & Colitis UK and C3, and by unrestricted educational grants from Abbvie (USA), Merck Sharp and Dohme (UK), NAPP Pharmaceuticals (UK), Pfizer (USA) and Celltrion Healthcare (South Korea). No funding bodies had any role in study design, data collection or analysis, writing or decision to submit for publication.

## 3 RESULTS

### 3.1 Participants

**FIGURE 1:**
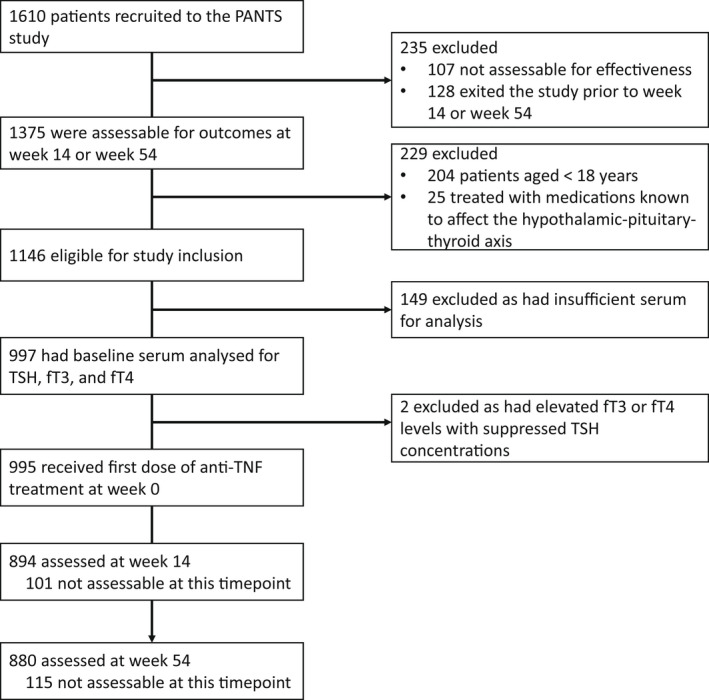
Study profile. Patients were not assessable when one of more key data items were missing. Abbreviations: TSH: thyroid‐stimulating hormone, fT3: free triiodothyronine, fT4: free thyroxine.

Overall, 86.9% (997/1146) adult patients who participated in PANTS were included: 549 (55.1%) were treated with infliximab (427 [42.8%] with originator infliximab and 122 [12.2%] with biosimilar CT‐P13) and 448 (44.9%) treated with adalimumab **(**Figure 1 **)**. We excluded 1.8% (25/1375) patients who were treated with medications known to affect the hypothalamic–pituitary–thyroid axis, including thyroxine, carbimazole, growth hormone and testosterone. 0.2% (2/997) patients had elevated fT3 or fT4 levels with suppressed TSH concentrations, suggestive of hyperthyroidism, and were excluded. No patients had hypothyroidism. No differences were seen in baseline characteristics between patients who were included in the study and in whom we did not have sufficient serum for analysis.

Differences between demographic and clinical characteristics of infliximab‐ and adalimumab‐treated patients are shown in Table 1. Similar to the whole cohort,^12^ there were significant demographic differences at baseline between the infliximab‐ and adalimumab‐treated patients, including in sex, age, ethnicity, disease behaviour and activity. At the initiation of anti‐TNF treatment, no differences were seen in the proportion of patients treated with immunomodulators or corticosteroids.

**TABLE 1:** Baseline demographic and clinical characteristics, stratified by anti‐TNF

Variable	Level	Adalimumab	Infliximab	Overall	*p*
*n*		448	549	997	
Sex	Female	49.33% (221/448)	55.74% (306/549)	52.86% (527/997)	0.048
	Male	50.67% (227/448)	44.26% (243/549)	47.14% (470/997)	
Ethnicity	White	95.54% (428/448)	92.35% (507/549)	93.78% (935/997)	0.034
	South Asian	2.68% (12/448)	2.91% (16/549)	2.81% (28/997)	
	Other	1.79% (8/448)	4.74% (26/549)	3.41% (34/997)	
Anti‐TNF	Adalimumab	100.00% (448/448)	0.00% (0/549)	44.93% (448/997)	<0.001
	Infliximab originator (Remicade)	0.00% (0/448)	77.78% (427/549)	42.83% (427/997)	
	Infliximab biosimilar (CT‐P13)	0.00% (0/448)	22.22% (122/549)	12.24% (122/997)	
Age at first dose of anti‐TNF		38.60 (28.81–51.01)	34.58 (26.03–47.32)	36.32 (27.30–49.21)	0.002
Disease duration		3.14 (0.77–11.62)	2.99 (0.72–10.13)	3.03 (0.73–10.68)	0.282
Montreal disease location	L1	32.05% (141/440)	31.74% (173/545)	31.88% (314/985)	0.167
	L2	21.36% (94/440)	27.16% (148/545)	24.57% (242/985)	
	L3	45.91% (202/440)	40.37% (220/545)	42.84% (422/985)	
	L4	0.68% (3/440)	0.73% (4/545)	0.71% (7/985)	
Montreal L4 modifier		3.86% (17/440)	4.95% (27/545)	4.47% (44/985)	0.442
Montreal disease behaviour	B1	56.88% (252/443)	56.80% (309/544)	56.84% (561/987)	0.005
	B2	36.79% (163/443)	31.07% (169/544)	33.64% (332/987)	
	B3	6.32% (28/443)	12.13% (66/544)	9.52% (94/987)	
Smoking history	Current	20.09% (89/443)	19.74% (107/542)	19.90% (196/985)	0.513
	Ex	35.67% (158/443)	32.47% (176/542)	33.91% (334/985)	
	Never	44.24% (196/443)	47.79% (259/542)	46.19% (455/985)	
Body mass index (kg /m^2^)		24.29 (21.48–28.26)	23.96 (20.85–28.20)	24.04 (21.03–28.25)	0.209
Baseline immunomodulator use	TRUE	52.01% (233/448)	55.56% (305/549)	53.96% (538/997)	0.278
Baseline steroid use	TRUE	28.79% (129/448)	29.87% (164/549)	29.39% (293/997)	0.727
C‐reactive protein (mg/L)		7.00 (2.00–14.00)	9.00 (3.00–22.00)	7.00 (3.00–18.00)	<0.001
Faecal calprotectin (μg/g)		317.00 (139.50–643.75)	404.00 (163.50–798.75)	350.50 (151.00–726.50)	0.006
Haemoglobin (g/L)		131.00 (121.00–142.00)	127.00 (116.00–138.00)	129.00 (118.00–139.00)	<0.001
Albumin (g/L)		39.00 (35.00–43.00)	39.00 (34.00–42.00)	39.00 (35.00–42.00)	0.083
Harvey Bradshaw Index		5.00 (3.00–8.00)	6.00 (3.00–9.00)	5.00 (3.00–9.00)	0.004

### 3.2 Factors associated with lower fT3/fT4 ratio

**FIGURE 2:**
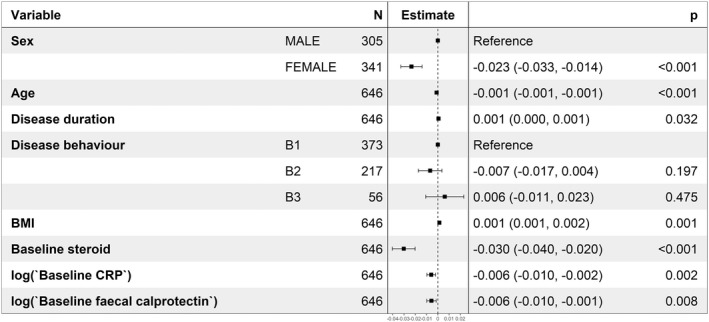
Forest plot showing the coefficients from a multivariable linear regression model of associations with fT3/fT4 ratio. The resultant values represent the change of fT3/fT4 ratio associated with each variable. Abbreviations: CRP = C‐reactive protein.

Serum fT3, fT4 and TSH concentrations (interquartile range [IQR]) were similar in infliximab‐ and adalimumab‐treated patients (fT3: infliximab 4.80 pmol/L [IQR 4.20–5.40] vs. adalimumab 4.90 pmol/L [IQR 4.40–5.50], *p* = 0.096; fT4: infliximab 15.60 pmol/L [IQR 14.10–17.10] vs. adalimumab 15.60 [IQR 14.30–17.50], *p* = 0.155; TSH: infliximab 1.34 [IQR 0.91–1.92] vs. adalimumab 1.39 [IQR 0.91–1.96], *p* = 0.287). Univariable analyses demonstrated that female sex, older age, lower BMI, shorter disease duration, higher CRP and faecal calprotectin concentrations and corticosteroid use at baseline, but not anti‐TNF type, smoking or immunomodulator use, were associated with lower fT3/fT4 ratio (Table 2). Multivariable linear regression analysis confirmed that female sex, higher CRP and faecal calprotectin concentrations and corticosteroid use were independently associated with lower fT3/fT4 ratio (Figure 2).

**TABLE 2:** Baseline demographic and clinical characteristics associated with fT3/fT4 ratio

Categorical variables				
Variable	Level	*n*	fT3/fT4 ratio	*p*
Sex	Female	526	0.30 (0.27–0.34)	<0.001
	Male	469	0.32 (0.28–0.36)	
Drug	Adalimumab	448	0.31 (0.28–0.35)	0.740
	Infliximab	547	0.31 (0.27–0.35)	
Smoker	Current smoker	196	0.31 (0.27–0.34)	0.364
	Non‐current smoker	787	0.31 (0.27–0.35)	
Corticosteroid use at baseline	Yes	293	0.28 (0.25–0.33)	<0.001
	No	702	0.32 (0.29–0.36)	
Immunomodulator use at baseline	Yes	537	0.31 (0.27–0.36)	0.156
	No	458	0.31 (0.27–0.35)	

Continuous variables		
Variable	Spearman's Rho (R)	*p*
Age	−0.12	<0.001
Disease duration	0.06	0.046
BMI	0.12	<0.001
CRP^a^	−0.10	0.002
Faecal calprotectin^a^	−0.12	0.001

Abbreviation: CRP, C‐reactive protein.

^a^ Variables were log‐transformed for analysis.

### 3.3 Association between fT3/fT4 ratio and clinical outcomes

**FIGURE 3:**
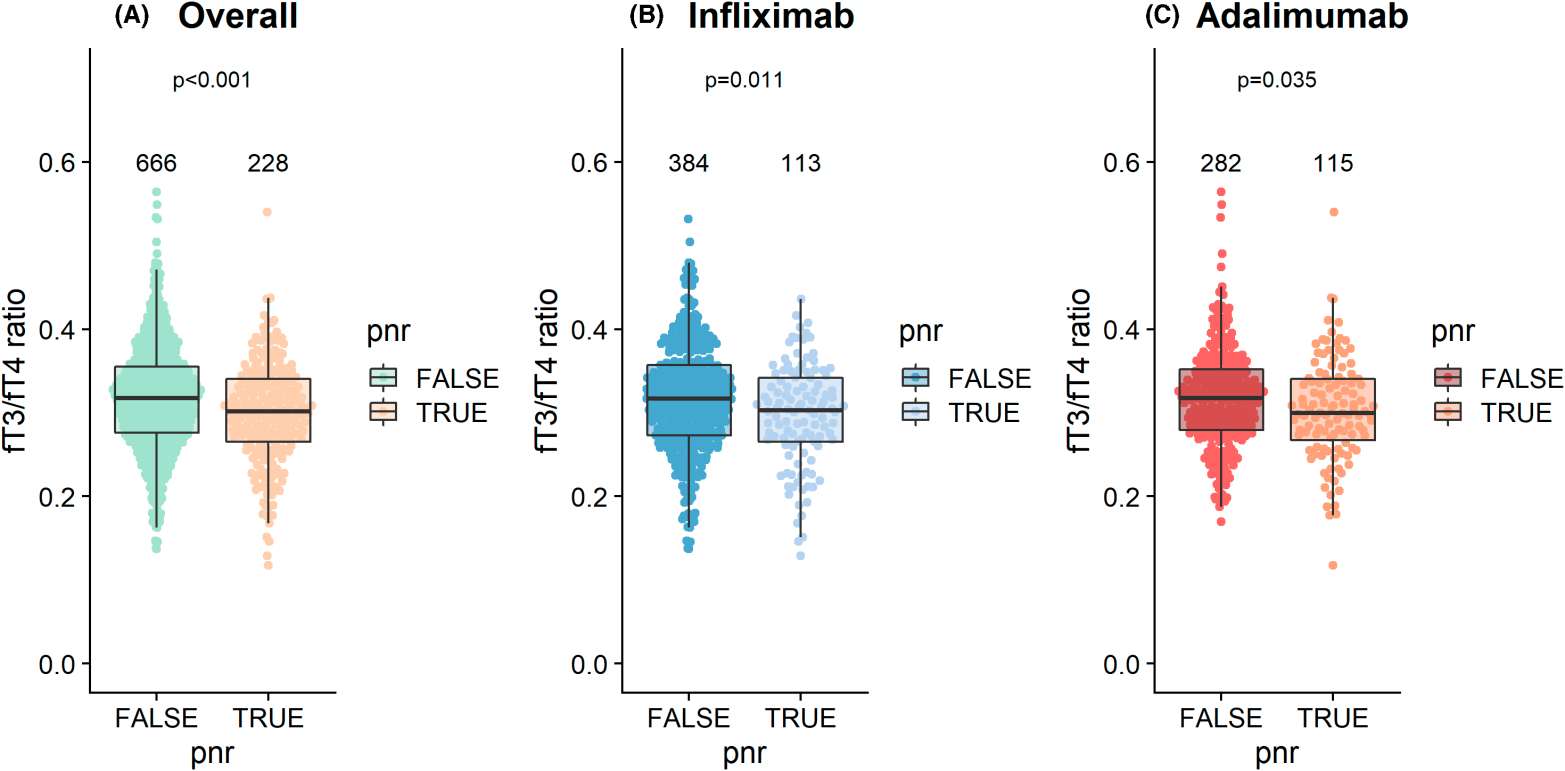
Beeswarm plot of fT3/fT4 ratio at baseline and primary non‐response at week 14, (A) combined cohort (B) infliximab‐treated patients, (C) adalimumab‐treated patients. The number of individuals tested for each group are shown in black at the top of each panel.

**FIGURE 4:**
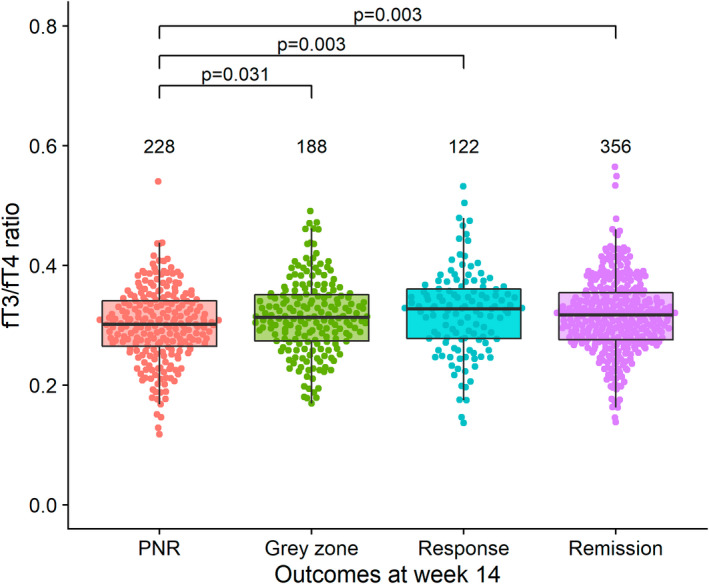
Beeswarm plot of fT3/fT4 ratio at baseline, stratified by outcome at week 14. The number of individuals tested for each group are shown in black at the top of each panel.

Overall, 89.8% (894/995) and 88.4% (880/995) patients included in the effectiveness analysis of the PANTS study at weeks 14 and 54, respectively, were included here. Of 894 patients who were assessable at week 14, 25.5% (228/894) patients experienced PNR, 21.0% (188/894) patients were classified as grey zone, 13.6% (122/894) patients as having had a response and 39.8% (356/894) patients were in remission. PNR occurred in 22.7% (113/497, 95% confidence interval [CI] 19.3–26.6%) of infliximab‐treated and 29.0% (115/397, 95% CI 24.7–33.6%) of adalimumab‐treated patients. Of 880 patients who were assessable at week 54, 65.1% (573/880) were classified as being in non‐remission, with no significant difference between infliximab‐ and adalimumab‐treated patients (*p* = 0.285). Univariable analyses across both anti‐TNF‐treated groups demonstrated that a lower fT3/fT4 ratio was associated with PNR (PNR: 0.30 [0.27–0.34] vs. no PNR 0.32 [0.28–0.36], *p* < 0.001) (Figures 3A and 4). Lower fT3/fT4 ratio remained significantly associated with PNR, when stratified by anti‐TNF drug (Figure 3B,C). No association was seen for baseline fT3/fT4 ratio and non‐remission at week 54.

**FIGURE 5:**
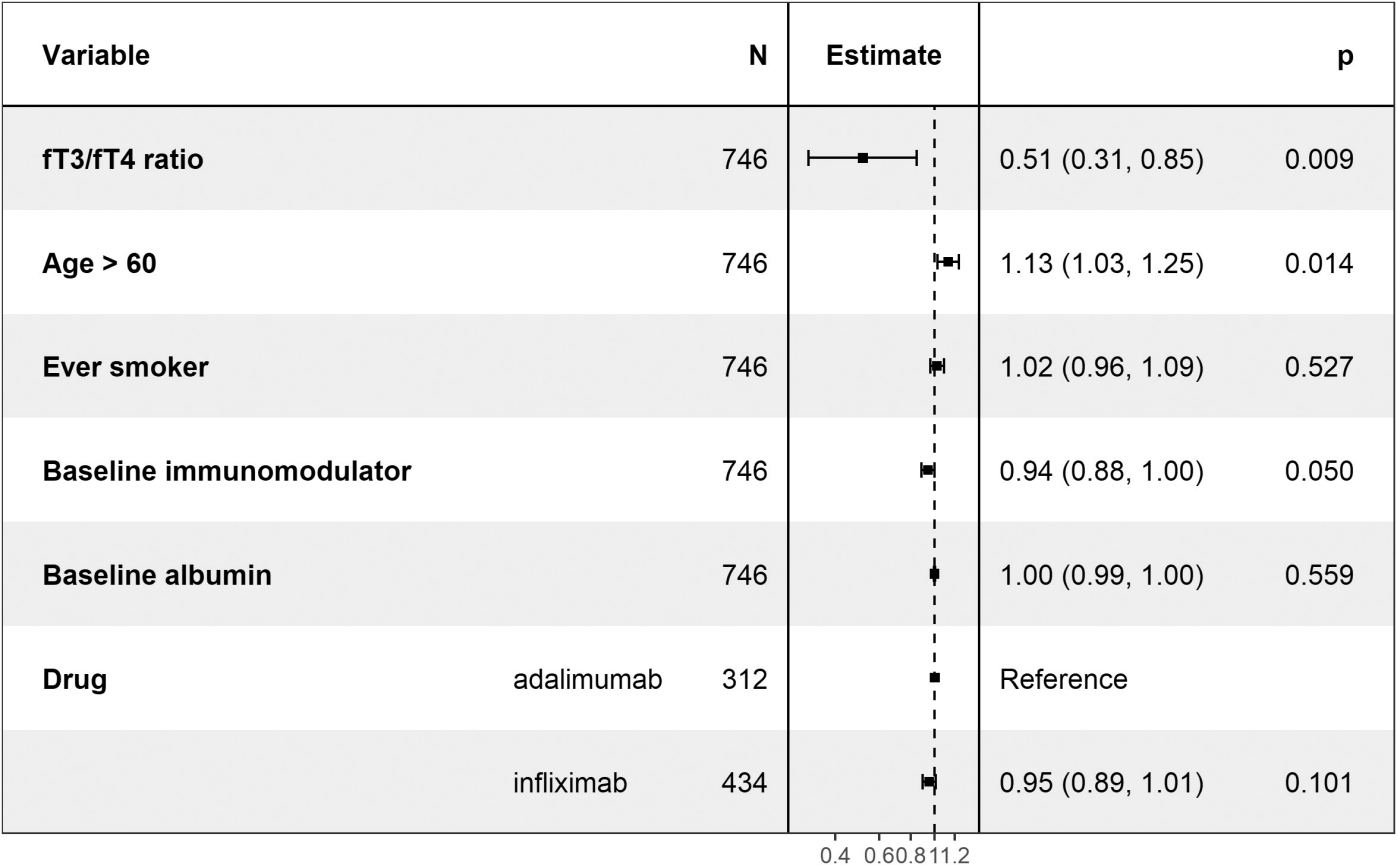
Forest plot showing the coefficients from a multivariable logistic regression model of associations with primary non‐response.

Multivariable logistic regression analyses confirmed that fT3/fT4 ratio was independently associated with PNR (odds ratio (OR) 0.51 [95% CI 0.31–0.85, *p* = 0.009) (Figure 5). When stratified by anti‐TNF and adjusted for variables known to be associated with PNR, low fT3/fT4 ratio remained associated with PNR for adalimumab‐, but not infliximab‐, treated patients (Figures S1 and S2).

Youden's method demonstrated that the optimal cut‐off threshold for baseline fT3/fT4 ratio to determine PNR at week 14 was 0.31, with an area under the curve of 0.57 (95% CI 0.54–0.61). The sensitivity and specificity were 0.62 (95% CI 0.41–0.74) 0.53 (95% CI 0.42–0.73), respectively. When incorporating anti‐TNF drug concentrations at week 14, in addition to fT3/fT4 ratio, we observed a marginal increase in AUC to 0.60 (95% CI 0.55–0.65).

### 3.4 Sensitivity analyses

We performed a sensitivity analysis assessing the association between fT3/fT4 ratio at baseline and faecal calprotectin at week 54. In a subset of 51% (451/880) of patients who had week 54 faecal calprotectin data, we found no correlation between fT3/fT4 ratio at baseline and, when assessing as a continuous variable, concentrations at week 54 (Rho = −0.079, *p* = 0.09). Furthermore, we found no difference in fT3/fT4 ratio at baseline between patients who had faecal calprotectin concentration >250 μg/g, representing active inflammation, and those who did not (0.31 [0.27–0.34] vs 0.31 [0.27–0.35], *p* = 0.39)

As steroid use was associated with fT3/fT4 ratio and is known to affect the hypothalamic–pituitary–thyroid axis, we performed a sensitivity analysis excluding patients treated with corticosteroids at baseline. Amongst 630 patients, there was no difference in fT3/fT4 ratio in those who experienced PNR compared with those who did not (PNR [128/630 patients]: 0.31 [IQR 0.28–0.35] vs. no PNR [502/630 patients]: 0.32 [IQR 0.29–0.36], *p* = 0.095). Lastly, we performed a sensitivity analysis restricting the cohort to those 60 years or over only. Among 105 patients, there was no difference in fT3/fT4 ratio in those who experienced PNR compared with those who did not (PNR [42/105 patients]: 0.27 [IQR 0.23–0.31] vs. no PNR [63/105 patients]: 0.29 [IQR 0.24–0.33], *p* = 0.190).

## 4 DISCUSSION

### 4.1 Key results

Lower baseline serum fT3/fT4 ratio was independently associated with female sex, higher inflammatory burden at baseline, and baseline corticosteroid use, and predicted PNR to anti‐TNF therapy at week 14, but not non‐remission or change in faecal calprotectin concentrations, at week 54. Overall, however, the diagnostic accuracy of baseline fT3/fT4 ratio to predict PNR to anti‐TNF treatment was modest, limiting its clinical utility.

### 4.2 Interpretation

This is the first large‐scale effort to examine the association between fT3/fT4 ratio and clinical outcomes in patients with IBD initiated on anti‐TNF therapy. Few previous studies have reported the prevalence of thyroid dysfunction or if serum fT3/fT4 levels influence the response to anti‐TNF therapy in patients with IBD.

Our observation that 1.8% and 0.2% patients were being treated for or had occult hypothyroidism, and hyperthyroidism respectively, is consistent with previous estimates of thyroid dysfunction in patients with IBD.^20^, ^21^ Up to 3.7% and 8.3% patients reportedly have concomitant hypothyroidism and hyperthyroidism, respectively, and rates are broadly similar to the background population.^7^

The pathophysiology underlying the non‐thyroidal illness syndrome is slowly being elucidated.

Relevant to patients with active IBD, proinflammatory cytokines and leptin reportedly have a critical role.^4^ They act centrally to reset release of thyroid releasing hormone from the paraventricular nucleus of the hypothalamus. Peripherally, they modulate serum thyroid hormone–binding protein levels and receptor expression and influence the activity of tissue deiodinases, which deactivate fT3 to 3,5‐diiodo‐L‐thyronine (T2) and fT4 to reverse triiodothyronine (rT3).^4^, ^22^

Bertani et al. showed that low serum fT3/fT4 ratio at initiation of infliximab or vedolizumab therapy predicted endoscopic outcomes at 54 weeks in a mixed cohort of patients with UC and Crohn's disease.^15^ Whilst we replicated the association with PNR to anti‐TNF therapies, we did not demonstrate an association between lower fT3/fT4 ratio and non‐remission, or changes in faecal calprotectin concentrations, after 1 year. Moreover, despite using a similar threshold, in our data the fT3/fT4 ratio lacked diagnostic accuracy to be clinically useful to predict PNR to anti‐TNF therapies at week 14.

There are a number of important differences in study design which may account for these discordant findings. Bertani et al. studied a mixed cohort of patients aged over 60 years with UC or Crohn's disease who were treated with either infliximab or vedolizumab, whereas we examined adults over 17 years with Crohn's disease and treated with an anti‐TNF drug only. When we restricted our analyses to patients over the age of 60 years, there was no longer an association between fT3/fT4 ratio on any of our predefined outcomes. Corticosteroid use at baseline is not reported in, or adjusted for, in the Bertani et al. study. Here, we have shown a negative association between corticosteroid use and fT3/fT4 ratio, like others have suggested.^23^, ^24^ Isolating the independent effect of corticosteroids on thyroid metabolism, however, remains challenging, largely due to underpowered studies with lack of adjustment for known confounders.^24^, ^25^, ^26^ Importantly, in our sensitivity analyses, we were unable to show an association between fT3/fT4 ratio and PNR in patients who were not treated with steroids at baseline. We acknowledge that corticosteroid use may reflect more active disease, and indeed was part of our definition of PNR, so the association between fT3/fT4 ratio and corticosteroids may be a combination of the direct effect of the corticosteroids on the hypothalamic–pituitary–thyroid axis and more severe IBD, as evidenced by raised CRP and higher faecal calprotectin concentrations.

Whether anti‐TNF treatment influences serum thyroid hormone levels is largely unknown. In a case series of 55 patients with IBD, Paschou et al. reported that fT4 concentrations reduced during anti‐TNF therapy, whilst fT3 and TSH levels were unchanged.^27^ Interestingly, they also observed higher than expected levels of thyroid autoimmunity. It is not clear whether this was due to a true increased risk of autoimmunity in patients with IBD, or whether they were detecting an excess of false positives because the anti‐TNF drugs interfered with the antithyroid antibody assay.

### 4.3 Limitations and generalizability

We acknowledge some important limitations of our work. We used pragmatic definitions of treatment failure combining corticosteroid use with clinical and biochemical markers of disease activity that are closely aligned to routine treatment targets. We accept that our data would have been strengthened by endoscopic outcomes as used by Bertani et al. However, in PANTS^12^ we observed a significant association between clinical outcomes at weeks 14 and week 54 and faecal calprotectin, which corelates closely with endoscopic findings; sensitivity analysis did not demonstrate an association between fT3/fT4 levels at baseline predicting changes in week 54 faecal calprotectin concentrations. We accept there was some missingness in our cohort, in particular, we were only able to include the adults enrolled in the PANTS study because of limited or exhausted stored serum in the paediatric patients who had lower volumes collected at each blood draw. Lastly, we analysed our stored serum several years after it was collected, against this having biased our results, median thyroid hormone levels were similar to the Bertani et al. study.

Our findings are likely to be generalisable to patients with Crohn's disease, and based on the Bertani et al. study report, to patients with UC. Anti‐TNF medications are used to treat a number of other immune‐mediated inflammatory diseases, which together affect about 5%–7% of Western populations including rheumatoid arthritis, ankylosing spondylitis, psoriatic arthritis, psoriasis, hidradenitis suppurativa and uveitis.^28^ Whether our findings are generalisable to other anti‐TNF drugs including adalimumab, certolizumab, golimumab and etanercept and other biologicals, across these other disease indications is unknown.

Predicting treatment response in patients with IBD is complex. Few of the so‐called precision medicine biomarkers to facilitate the right drug, to the right patient, at the right time have translated to clinical care.^14^, ^29^, ^30^, ^31^ In part, this is because of their relatively modest effect size and the challenges of clinical translation of the basic science. The initial findings of the Bertani et al. study were exciting, not least because the fT3/fT4 ratio is a physiological barometer of the complex adaptations that occur as a consequence of inflammation in patients with IBD and because thyroid hormone testing is inexpensive and already set‐up in most hospitals. Based on our findings however, further work using endoscopic outcomes by disease and biologic type is needed to confirm or refute the usefulness of the fT3/fT4 ratio to predict anti‐TNF treatment outcomes. Our results do not suggest fT3/fT4 ratio as a predictor of anti‐TNF response is clinically useful, however, it may have a role in a larger panel including pharmacokinetic variables such as drug concentration, and emergent molecular biomarkers that may be clinically significant, such as Oncostatin M.^30^, ^31^

## 5 CONCLUSIONS

Lower baseline serum fT3/fT4 ratio was associated with female sex, higher inflammatory burden at baseline, and corticosteroid use and predicted PNR to anti‐TNF treatment at week 14, but not non‐remission, or changes in faecal calprotectin concentrations at week 54. Overall, serum fT3/fT4 ratio to predict PNR lacked diagnostic accuracy and is unlikely to be a clinically useful predictor.

## AUTHOR CONTRIBUTIONS

Simeng Lin: Conceptualization (equal); data curation (equal); formal analysis (equal); investigation (equal); methodology (equal); writing – original draft (equal); writing – review and editing (equal). Neil Chanchlani: Conceptualization (equal); data curation (equal); formal analysis (equal); investigation (equal); methodology (equal); writing –original draft (equal); writing –review and editing (equal); Isabel Carbery: Conceptualization (equal); data curation (equal); formal analysis (equal); investigation (equal); writing – original draft (equal); writing – review and editing (equal). Malik Janjua: Data curation (equal); writing – review and editing (supporting). Rachel Nice: Data curation (equal); investigation (equal); writing – review and editing (equal). Timothy J McDonald: Data curation (equal). Claire Bewshea: Project administration (equal). Nicholas Alexander Kennedy: Data curation (equal); formal analysis (equal); investigation (equal); methodology (equal); writing – review and editing (equal). Tariq Ahmad: Conceptualization (equal); data curation (equal); funding acquisition (equal); investigation (equal); methodology (equal); supervision (equal); writing – review and editing (equal). Christian P Selinger: Conceptualization (equal); data curation (equal); formal analysis (equal); funding acquisition (equal); investigation (equal); methodology (equal); supervision (equal); writing – original draft (equal); writing – review and editing (equal).

## CONFLICT OF INTEREST

Dr. Lin reports non‐financial support from Pfizer, non‐financial support from Ferring, outside the submitted work. Dr. Kennedy reports grants from F. Hoffmann‐La Roche AG, grants from Biogen Inc, grants from Celltrion Healthcare, grants from Galapagos NV, non‐financial support from Immundiagnostik, grants and non‐financial support from AbbVie, grants and personal fees from Celltrion, personal fees and non‐financial support from Janssen, personal fees from Takeda, personal fees and non‐financial support from Dr Falk, outside the submitted work.

Prof. Ahmad reports grants and non‐financial support from F. Hoffmann‐La Roche AG, grants from Biogen Inc, grants from Celltrion Healthcare, grants from Galapagos NV, non‐financial support from Immundiagnostik, personal fees from Biogen inc, grants and personal fees from Celltrion Healthcare, personal fees and non‐financial support from Immundiagnostik, personal fees from Takeda, personal fees from ARENA, personal fees from Gilead, personal fees from Adcock Ingram Healthcare, personal fees from Pfizer, personal fees from Genentech, non‐financial support from Tillotts, outside the submitted work. Dr Selinger has received unrestricted research grants from Warner Chilcott, Janssen and AbbVie, has provided consultancy to Warner Chilcott, Dr Falk, AbbVie, Takeda, Fresenius Kabi, Arena, Galapagos, Celltrion, Norgine and Janssen, and had speaker arrangements with Warner Chilcott, Norgine, Galapagos, Fresenius Kabi, Celtrion, Dr Falk, AbbVie, MSD, Pfizer and Takeda. Dr. Goodhand reports grants from F. Hoffmann‐La Roche AG, grants from Biogen Inc, grants from Celltrion Healthcare, grants from Galapagos NV, non‐financial support from Immundiagnostik, outside the submitted work. The following authors have nothing to declare: Neil Chanchlani, Isabel Carbery, Malik Janjua, Rachel Nice, Timothy J McDonald and Claire Bewshea.

## AUTHORSHIP

*Guarantor of the article*: James R. Goodhand.

## DATA AVAILABILITY STATEMENT

Individual participant de‐identified data that underlie the results reported in this article will be available immediately after publication for a period of 5 years. The data will be made available to investigators whose proposed use of the data has been approved by an independent review committee. Analyses will be restricted to the aims in the approved proposal. Proposals should be directed to james.goodhand@nhs.net. To gain access data requestors will need to sign a data access agreement.

## Supporting information


Data S1
