# Supplementary material for: Understanding anti‐TNF treatment failure: does serum triiodothyronine‐to‐thyroxine (T3/T4) ratio predict therapeutic outcome to anti‐TNF therapies in biologic‐naïve patients with active luminal Crohn's disease?
Source: Aliment Pharmacol Ther. 2022 Jun 29;56(5):783–93. doi: 10.1111/apt.17089 (PMC9540440; doi:10.1111/apt.17089)

**Supplementary information** for “Does serum triiodothyronine-to-thyroxine (T3/T4) ratio predict therapeutic outcome to anti-TNF therapies in biologic-naïve patients with active luminal Crohn’s disease?

Table of Contents

[Supplementary Table 1: PANTS consortium 2](#_Toc102640175)

[Supplementary Figure 1: Forest plot showing the coefficients from a multivariable logistic regression model of associations with primary non-response, and adjusted for variables known to be associated, stratified by anti-TNF (infliximab) 9](#_Toc102640176)

[Supplementary Figure 2: Forest plot showing the coefficients from a multivariable logistic regression model of associations with primary non-response, and adjusted for variables known to be associated, stratified by anti-TNF (adalimumab) 10](#_Toc102640177)

# Supplementary Table 1: PANTS consortium

All UK gastroenterologists were invited to participate in the PANTS study which was promoted through the UK National Institute for Health Service Research (NIHR) and the British Society of Gastroenterology (BSG).

| **Hospital or Trust name** | **City** | **Name** | **Job Title** |
| --- | --- | --- | --- |
| Tameside Hospital NHS Foundation Trust | Ashton U Lyne | Dr Vinod Patel | Consultant Gastroenterologist |
| Basildon and Thurrock University Hospitals NHS Foundation Trust | Basildon | Dr Zia Mazhar | Consultant Gastroenterologist |
| Hampshire Hospitals NHS Foundation Trust | Basingstoke | Dr Rebecca Saich | Consultant Gastroenterologist |
| Royal United Hospital | Bath | Dr Ben Colleypriest | Consultant Gastroenterologist |
| Ulster Hospital | Belfast | Dr Tony C Tham | Consultant Gastroenterologist |
| University Hospital's Birmingham NHS Foundation Trust | Birmingham | Dr Tariq H Iqbal | Consultant Gastroenterologist |
| East Lancashire NHS Teaching Trust | Blackburn | Dr Vishal Kaushik | Consultant Gastroenterologist |
| Blackpool Teaching Hospitals NHS Foundation Trust | Blackpool | Dr Senthil Murugesan | Consultant Gastroenterologist |
| Bolton NHS Trust | Bolton | Dr Salil Singh | Consultant Gastroenterologist |
| Royal Bournemouth Hospital | Bournemouth | Dr Sean Weaver | Consultant Gastroenterologist |
| Bradford Teaching Hospitals Foundation Trust - (St Lukes Hospital &Bradford Royal Infirmary) | Bradford | Dr Cathryn Preston | Consultant Gastroenterologist |
| Brighton and Sussex University Hospitals NHS Trust | Brighton | Dr Assad Butt | Paediatric Consultant Gastroenterologist |
| Brighton and Sussex University Hospitals NHS Trust | Brighton | Dr Melissa Smith | Consultant Gastroenterologist |
| University Hospitals Bristol NHS Foundation Trust | Bristol | Dr Dharamveer Basude | Consultant Paediatric Gastroenterologist |
| University Hospitals Bristol NHS Foundation Trust | Bristol | Dr Amanda Beale | Consultant Gastroenterologist |
| Frimley Park Hospital NHS Foundation Trust | Camberley | Dr Sarah Langlands | Consultant Gastroenterologist |
| Frimley Park Hospital NHS Foundation Trust | Camberley | Dr Natalie Direkze | Consultant gastroenterologist |
| Cambridge University Hospitals NHS Foundation Trust | Cambridge | Dr Miles Parkes | Consultant Gastroenterologist |
| Cambridge University Hospitals NHS Foundation Trust | Cambridge | Dr Franco Torrente | Consultant Paediatric Gastroenterologist |
| Cambridge University Hospitals NHS Foundation Trust | Cambridge | Dr Juan De La Revella Negro | Research fellow |
| North Cumbria University Hospitals NHS Trust | Carlisle | Dr Chris Ewen MacDonald | Consultant Gastroenterologist |
| Ashford & St Peter's Hospitals NHS Foundation Trust | Chertsey | Dr Stephen M Evans | Consultant Gastroenterologist |
| St Peter's Hospital | Chertsey | Dr Anton V J Gunasekera | Consultant Gastroenterologist |
| Ashford & St Peter's Hospitals NHS Foundation Trust | Chertsey | Dr Alka Thakur | Paediatric Consultant |
| Chesterfield Royal NHS Foundation Trust | Chesterfield | Dr David Elphick | Consultant Gastroenterologist |
| Colchester Hospital University NHS Foundation Trust | Colchester | Dr Achuth Shenoy | Consultant Gastroenterologist |
| University Hospitals Coventry and Warwickshire NHS Trust | Coventry | Prof Chuka U Nwokolo | Consultant Gastroenterologist |
| County Durham and Darlington NHS Foundation Trust | Darlington | Dr Anjan Dhar | Consultant Gastroenterologist & Hon. Clinical Lecturer |
| Derby Hospital NHS Foundation NHS Trust | Derby | Dr Andrew T Cole | Consultant Gastroenterologist |
| Doncaster and Bassetlaw Hospitals NHS Foundation Trust | Doncaster | Dr Anurag Agrawal | Consultant Gastroenterologist |
| Dorset County Hospital NHS Foundation Trust | Dorchester | Dr Stephen Bridger | Consultant Gastroenterologist |
| Dorset County Hospitals Foundation Trust | Dorchester | Dr Julie Doherty | Paediatric Consultant |
| Dudley Group NHS Foundation Trust | Dudley | Dr Sheldon C Cooper | Consultant Gastroenterologist |
| Russells Hall Hospital, The Dudley Group NHS Foundation Trust | Dudley | Dr Shanika de Silva | Consultant Gastroenterologist |
| Ninewells Hospital & Medical School | Dundee | Dr Craig Mowat | Consultant Gastroenterologist |
| East Sussex Healthcare Trust | Eastborne | Dr Phillip Mayhead | Consultant Gastroenterologist |
| NHS Lothian | Edinburgh | Dr Charlie Lees | Consultant Gastroenterologist and Honorary Senior Lecturer |
| NHS Lothian | Edinburgh | Dr Gareth Jones | Research fellow |
| Royal Devon and Exeter NHS Foundation Trust | Exeter | Dr Tariq Ahmad | Consultant Gastroenterologist |
| Royal Devon and Exeter NHS Foundation Trust | Exeter | Dr James W Hart | Consultant Paediatrician |
| Glasgow Royal Infirmary | Glasgow | Dr Daniel R Gaya | Consultant Gastroenterologist |
| Royal Hospital for Children | Glasgow | Prof Richard K Russell | Consultant Paediatric Gastroenterologist |
| Royal Hospital for Children | Glasgow | Dr Lisa Gervais | Research fellow |
| Gloucestershire Hospitals NHS Trust | Gloucester | Dr Paul Dunckley | Consultant Gastroneterologist |
| United Lincolnshire Hospitals NHS Trust | Grantham | Dr Tariq Mahmood | Consultant Gastroenterologist |
| James Paget University Hospitals NHS Foundation Trust | Great Yarmouth | Dr Paul J R Banim | Consultant Gastroneterologist |
| Calderdale and Huddersfield NHS Trust | Halifax | Dr Sunil Sonwalkar | Consultant Gastroenterologist |
| Princess Alexandra Hospital NHS Trust | Harlow | Dr Deb Ghosh | Consultant Gastroenterologist |
| Princess Alexandra Hospital NHS Trust | Harlow | Dr Rosemary H Phillips | Consultant Gastroenterologist |
| Hull and East Yorkshire NHS Trust | Hull | Dr Amer Azaz | Paediatric Consultant Gastroenterologist |
| Hull and East Yorkshire NHS Trust | Hull | Dr Shaji Sebastian | Consultant Gastroenterologist |
| Airedale NHS Foundation Trust | Keighley | Dr Richard Shenderey | Consultant Gastroenterologist |
| Crosshouse Hospital | Kilmarnock | Dr Lawrence Armstrong | Consultant Paediatrician |
| Crosshouse Hospital | Kilmarnock | Dr Claire Bell | Research fellow |
| The Queen Elizabeth Hospital NHS Foundation Trust | Kings Lynn | Dr Radhakrishnan Hariraj | Consultant Gastroenterologist |
| Kingston Hospital NHS Trust | Kingston upon Thames | Dr Helen Matthews | Consultant Gastroenterologist |
| NHS Fife | Kirkcaldy | Dr Hasnain Jafferbhoy | Consultant Gastroenterologist |
| Leeds Teaching Hospitals NHS Trust | Leeds | Dr Christian P Selinger | Consultant Gastroenterologist |
| Leeds Teaching Hospitals NHS Trust | Leeds | Dr Veena Zamvar | Paediatric Consultant Gastroenteorlogist |
| University Hospitals of Leicester NHS Trust | Leicester | Prof John S De Caestecker | Consultant Gastroenterologist |
| University Hospitals of Leicester NHS Trust | Leicester | Dr Anne Willmott | Paediatric Consultant Gastroenterologist |
| Mid Cheshire Hospitals NHS Foundation Trust | Leighton | Mr Richard Miller | Research Nurse |
| United Lincolnshire Hospitals NHS Trust | Lincoln | Dr Palani Sathish Babu | Consultant Gastroenterologist |
| Alder Hey Childrens Hospital | Liverpool | Dr Christos Tzivinikos | Consultant Paediatric Gastroenterologist |
| University College London Hospitals NHS Foundation Trust | London | Dr Stuart L Bloom | Consultant Gastroenterologist |
| Kings College Hospital NHS Foundation Trust | London | Dr Guy Chung-Faye | Consultant Gastroenterologist |
| Royal London Childrens Hospital, Barts Health NHS Trust | London | Prof Nicholas M Croft | Paediatric Consultant Gastroenterologist |
| Chelsea & Westminster Hospital | London | Dr John ME Fell | Consultant Paediatric Gastroenterologist |
| Chelsea and Westminster Hospital NHS Foundation | London | Dr Marcus Harbord | Consultant Gastroenterologist |
| North West London Hospitals NHS Trust | London | Dr Ailsa Hart | Consultant Gastroenterologist |
| Kings College Hospital NHS Foundation Trust | London | Dr Ben Hope | Consultant Paediatrician |
| Guys & St Thomas' NHS Foundation Trust | London | Dr Peter M Irving | Consultant Gastroenterologist |
| Barts and The London NHS Trust | London | Prof James O Lindsay | Consultant Gastroenterologist |
| Guy's and St Thomas' NHS trust | London | Dr Joel E Mawdsley | Gastroenterology Consultant |
| Lewisham and Greenwich Healthcare NHS Trust | London | Dr Alistair McNair | Consultant Gastroenterologist |
| Chelsea and Westminster Hospital NHS Foundation | London | Dr Kevin J Monahan | Consultant Gastroenterologist |
| Royal Free London NHS Foundation Trust | London | Dr Charles D Murray | Consultant Gastroenterologist |
| Imperial College Healthcare NHS Trust | London | Prof Timothy Orchard | Consultant Gastroenterologist |
| St George's Healthcare NHS Trust | London | Dr Thankam Paul | Paediatric Consultant Gastroenterologist |
| St George's Healthcare NHS Trust | London | Dr Richard Pollok | Reader and Consultant Gastroenterologist |
| Great Ormond Street Hospital for Children NHS Foundation Trust | London | Dr Neil Shah | Consultant Gastroenterologist |
| North West London Hospitals NHS Trust | London | Dr Sonia Bouri | Research fellow |
| The Luton & Dunstable University Hospital | Luton | Dr Matt W Johnson | Consultant Gastroenterologist |
| Luton and Dunstable Hospital Foundation Trust | Luton | Dr Anita Modi | Paediatric Consultant with Allergy and Gastroenterology interest |
| The Luton & Dunstable University Hospital | Luton | Dr Kasamu Dawa Kabiru | Research fellow |
| Maidstone and Tunbridge Wells NHS Trust | Maidstone | Dr B K Baburajan | Consultant Gastroenterologist |
| Maidstone and Tunbridge Wells NHS Trust | Maidstone | Prof Bim Bhaduri | Paediatric Consultant Gastroenterologist |
| Manchester University Hospitals NHS Foundation Trust | Manchester | Dr Andrew Adebayo Fagbemi | Consultant Gastroenterologist |
| Central Manchester University Hospitals NHS Foundation Trust | Manchester | Dr Scott Levison | Consultant Gastroenterologist |
| The Pennine Acute Hospitals NHS Trust | Manchester | Dr Jimmy K Limdi | Consultant Gastroenterologist |
| Manchester University NHS Foundation Trust, Wythenshawe Hospital | Manchester | Dr Gill Watts | Consultant Gastroenterologist |
| Sherwood Forest Hospitals NHS Foundation Trust | Mansfield | Dr Stephen Foley | Consultant Gastroenterologist |
| South Tees Hospital NHS Foundation Trust | Middlesbrough | Dr Arvind Ramadas | Consultant Gastroenterologist |
| Milton Keynes Hospital NHS Foundation Trust | Milton Keynes | Dr George MacFaul | Consultant Gastroenterologist |
| Newcastle Upon Tyne Hospital Trust | Newcastle | Dr John Mansfield | Consultant Gastroenterologist |
| Isle of Wight NHS Foundation Trust | Newport | Dr Leonie Grellier | Consultant Gastroenterologist |
| Norfolk & Norwich University Hospital NHS Foundation Trust | Norwich | Dr Mary-Anne Morris | Consultant Paediatric Gastroenterologist |
| Norfolk & Norwich University Hospital NHS Foundation Trust | Norwich | Dr Mark Tremelling | Consultant Gastroenterologist |
| Nottingham University Hospitals NHS Trust | Nottingham | Prof Chris Hawkey | Consultant Gastroenterologist |
| Nottingham University Hospitals NHS Trust | Nottingham | Dr Sian Kirkham | Consultant Paediatric Gastroenterologist |
| Nottingham University Hospitals NHS Trust | Nottingham | Dr Charles PJ Charlton | Consultant gastroenterologist |
| Oxford University Hospitals NHS Foundation Trust | Oxford | Dr Astor Rodrigues | Paediatric Consultant Gastroenterologist |
| Oxford University Hospitals NHS Trust | Oxford | Prof Alison Simmons | Consultant Gastroenterologist |
| Plymouth Hospitals NHS Trust | Plymouth | Dr Stephen J Lewis | Consultant Gastroenterologist |
| Poole Hospital NHS Foundation Trust | Poole | Dr Jonathon Snook | Consultant Gastroenterologist |
| Poole Hospital NHS Foundation Trust | Poole | Dr Mark Tighe | Paediatric Consultant with interest in Oncology and Gastroenterology |
| Portsmouth Hospitals NHS Trust | Portsmouth | Dr Patrick M Goggin | Consultant Gastroenterologist |
| Royal Berkshire NHS Foundation Trust | Reading | Dr Aminda N De Silva | Consultant Gastroenterologist |
| Salford Royal NHS Foundation Trust | Salford | Prof Simon Lal | Consultant Gastroenterologist |
| Shrewsbury and Telford Hospital NHS Trust | Shrewsbury | Dr Mark S Smith | Consultant Gastroenterologist |
| South Tyneside NHS Foundation Trust | South Shields | Dr Simon Panter | Consultant Gastroenterologist |
| Southampton University Hospitals NHS Trust | Southampton | Dr JR Fraser Cummings | Consultant Gastroenterologist |
| Southampton University Hospitals NHS Trust | Southampton | Dr Suranga Dharmisari | Research fellow |
| East and North Herts NHS Trust | Stevenage | Dr Martyn Carter | Consultant Gastroenterologist |
| NHS Forth Valley | Stirling | Dr David Watts | Consultant Gastroenterologist |
| Stockport NHS foundation Trust | Stockport | Dr Zahid Mahmood | Consultant Gastroenterologist |
| North Tees and Hartlepool NHS Foundation Trust | Stockton | Dr Bruce McLain | Paediatric Consultant Gastroenterologist |
| University Hospitals of North Staffordshire | Stoke-on Trent | Dr Sandip Sen | Consultant Gastroenterologist |
| University Hospitals of North Midlands NHS Trust | Stoke-on-Trent | Dr Anna J Pigott | Consultant Paediatric Gastroenterologist |
| City Hospitals Sunderland NHS Foundation Trust | Sunderland | Dr David Hobday | Consultant Gastroenterologist |
| Taunton and Somerset NHS Foundation Trust | Taunton | Dr Emma Wesley | Consultant Gastroenterologist |
| South Devon Healthcare NHS Foundation Trust | Torquay | Dr Richard Johnston | Consultant Gastroenterologist |
| South Devon Healthcare NHS Foundation Trust | Torquay | Dr Cathryn Edwards | Consultant gastroenterologist |
| Royal Cornwall Hospitals NHS Trust | Truro | Dr John Beckly | Consultant Gastroenterologist |
| Mid Yorkshire Hospitals NHS Trust | Wakefield | Dr Deven Vani | Consultant Physician & Gastroenterologist |
| Warrington& Halton NHS Foundation | Warrington | Dr Subramaniam Ramakrishnan | Consultant Gastroenterologist |
| West Hertfordshire Hospitals NHS Trust | Watford | Dr Rakesh Chaudhary | Consultant Gastroenterologist |
| Sandwell and West Birmingham Hospitals NHS Trust | West Bromwich | Dr Nigel J Trudgill | Consultant Gastroenterologist |
| Sandwell and West Birmingham Hospitals NHS Trust | West Bromwich | Dr Rachel Cooney | Consultant gastroenterologist |
| Weston Area Health NHS Trust | Weston-Super-Mare | Dr Andy Bell | Consultant Gastroenterologist |
| Royal Albert Edward Infirmary, Wrightington, Wigan & Leigh NHS Foundation Trust | Wigan | Dr Neeraj Prasad | Consultant Gastroenterologist |
| Hampshire Hospitals NHS Foundation Trust | Winchester | Dr John N Gordon | Consultant Gastroenterologist |
| Royal Wolverhampton Hospitals NHS Trust | Wolverhampton | Prof Matthew J Brookes | Consultant Gastroenterologist |
| Western Sussex Hospitals NHS Trust | Worthing | Dr Andy Li | Consultant Gastroenterologist |
| Yeovil District Hospital NHS Foundation Trust | Yeovil | Dr Stephen Gore | Consultant Gastroenterologist |

# Supplementary Figure 1: Forest plot showing the coefficients from a multivariable logistic regression model of associations with primary non-response, and adjusted for variables known to be associated, stratified by anti-TNF (infliximab)


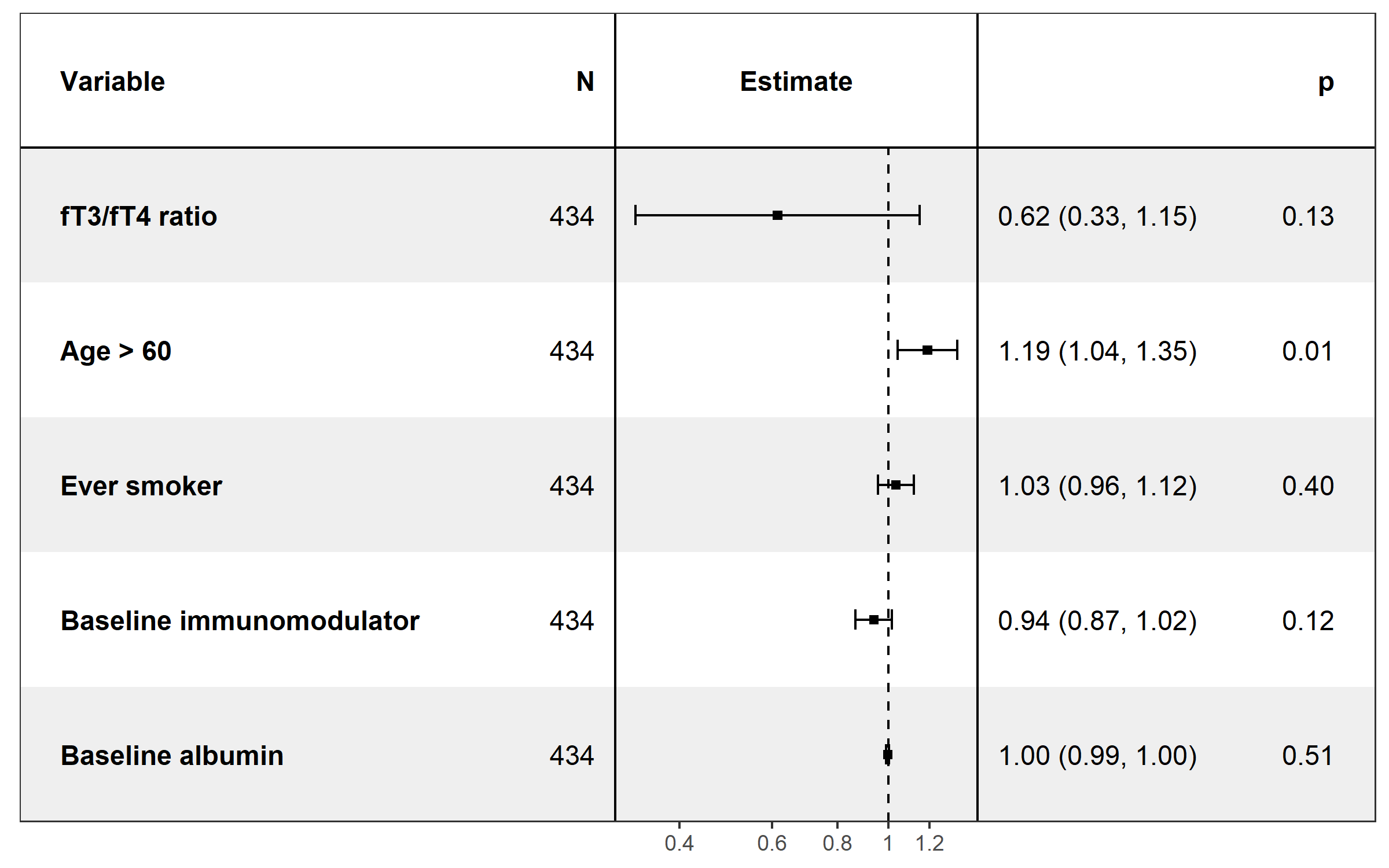


# Supplementary Figure 2: Forest plot showing the coefficients from a multivariable logistic regression model of associations with primary non-response, and adjusted for variables known to be associated, stratified by anti-TNF (adalimumab)


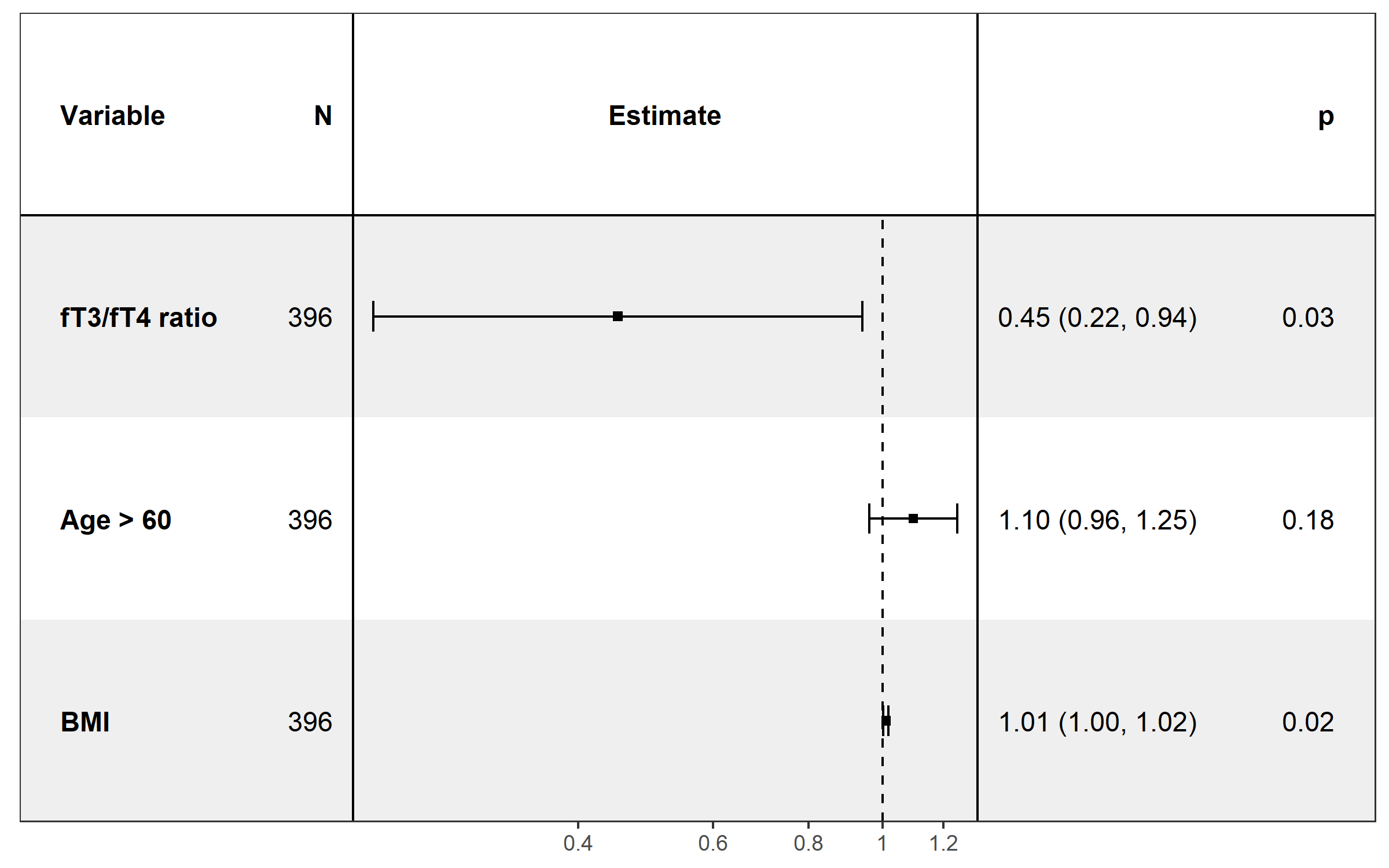

Supplement: Supplementary file 1 — Data S1 [file APT-56-783-s001.docx]
